# Effectiveness of Pulsed Radiofrequency of the Greater Occipital Nerve in Cervical Myofascial Pain Syndrome: A Retrospective Observational Study

**DOI:** 10.1155/prm/4382163

**Published:** 2026-08-25

**Authors:** Victor Cruz Guisado, Laura Garcia Aparicio, Estefania Guisado Fernandez, Enrique Arias Rosado, Maria Rodriguez Aguilar

**Affiliations:** ^1^ Department of Physical Medicine and Rehabilitation, AGS Jerez, Costa Noroeste y Sierra de Cádiz, Cádiz, Andalusia, Spain; ^2^ Departamento Materno-Infantil Y Radiología, Universidad De Cádiz, Cádiz, Andalusia, Spain, uca.es; ^3^ Grade in Medicine, University of Cádiz, Cádiz, Andalusia, Spain, uca.es

**Keywords:** cervical myofascial pain, greater occipital nerve, neck pain, neuromodulation, pulsed radiofrequency

## Abstract

**Background:**

Cervical myofascial pain syndrome (CMPS) is a common cause of neck pain and disability, often refractory to conservative management. Pulsed radiofrequency (PRF) of the greater occipital nerve (GON) has emerged as a minimally invasive neuromodulatory treatment for chronic cervicogenic pain.

**Objective:**

To evaluate the effectiveness and safety of ultrasound‐guided PRF of the GON in patients with cervical myofascial pain refractory to conventional therapy.

**Methods:**

A retrospective observational study was conducted, including 14 patients diagnosed with cervical MPS treated with PRF of the GON at the Hospital Universitario de Jerez de la Frontera. Pain intensity, assessed using the Visual Analog Scale (VAS), was evaluated before and after the intervention. Data were analyzed using Wilcoxon and correlation analyses.

**Results:**

Mean pain scores significantly decreased from 7.93 (SD = 1.77) to 4.57 (SD = 2.38) postintervention (mean difference = 3.36, *p* = 0.00375). This reduction exceeded the minimal clinically important difference of three points on the VAS. No significant associations were found between demographic variables and treatment response. No major adverse effects were reported.

**Conclusion:**

PRF of the GON appears to be an effective and safe therapeutic option for patients with refractory cervical CMPS, resulting in a significant pain reduction. Further prospective studies with larger samples are warranted.

## 1. Introduction

Neck pain is one of the most prevalent musculoskeletal conditions, second only to low back pain as a cause of spinal pain and disability worldwide [1, 2]. Cervical myofascial pain syndrome (CMPS) is a frequent etiology of chronic neck pain [3, 4], characterized by the presence of trigger points (TrPs) in the cervical musculature and associated referred pain patterns [5–7]. MPS is defined as musculoskeletal pain, characterized by myofascial TrPs, reduced mobility, and a referral pattern of pain [8, 9].

Cervical myofascial pain syndrome is primarily a clinical diagnosis characterized by regional nonradicular neck pain associated with active myofascial TrPs located in cervical and periscapular muscles. Diagnosis is based on physical examination findings, including the presence of palpable taut bands; hypersensitive nodules; reproduction of the patient’s typical pain upon manual palpation; characteristic referred pain patterns; and restriction of cervical range of motion, in the absence of neurological deficits [3–6, 8]. Imaging studies are not diagnostic of myofascial pain but are used to exclude structural causes of cervical pain [3, 4, 8].

Traditional treatments include physical therapy [2], pharmacologic interventions, and local injections, such as TrPs infiltrations [3, 10]. However, many patients remain symptomatic despite comprehensive conservative management [2].

Recent advances in interventional pain medicine have led to the exploration of pulsed radiofrequency (PRF) as a neuromodulatory technique capable of altering nociceptive transmission without causing neural destruction [11]. The greater occipital nerve (GON), a key sensory branch of the C2 dorsal ramus, plays a pivotal role in occipital and upper cervical pain syndromes [12–15].

While PRF of the GON is established in conditions like occipital neuralgia [16–21], its use in related headache and cervicogenic pain disorders is an active area of research [22]. Evidence for its specific use in cervical MPS remains limited. This study aims to assess the efficacy and safety of ultrasound‐guided PRF applied to the GON in patients with refractory cervical MPS.

## 2. Methods

### 2.1. Study Design and Population

This retrospective observational study analyzed medical records of patients treated between June 2017 and June 2023 at the Department of Physical Medicine and Rehabilitation, Hospital Universitario de Jerez de la Frontera (Cádiz, Spain). Inclusion criteria were: (1) clinical diagnosis of CMPS or nonradicular cervicalgia; (2) refractoriness to conservative treatment; (3) prior positive diagnostic block of the GON; and (4) treatment with ultrasound‐guided PRF. Exclusion criteria included cervicogenic headache, radiculopathy, fibromyalgia, tumor, infection, or previous cervical surgery.

The diagnosis of CMPS was established clinically by experienced rehabilitation physicians, based on chronic nonradicular cervical pain, identification of active myofascial TrPs in cervical musculature, reproduction of the patient’s usual pain during physical examination, associated referred pain patterns, and functional limitation of cervical mobility. Cervical magnetic resonance imaging or other imaging studies were reviewed when available to exclude structural pathology such as disc herniation, spinal canal stenosis, tumor, infection, or other conditions that could explain the symptoms.

**FIGURE 1:**
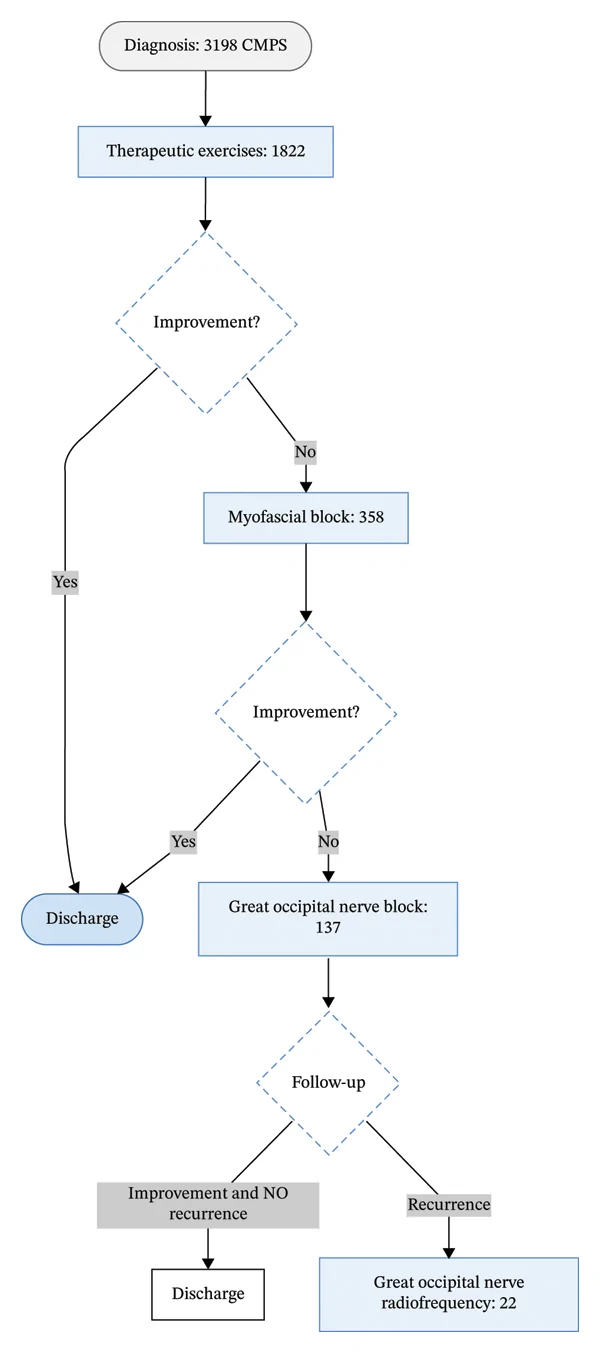
Flow diagram of patient selection and study protocol. Of all patients evaluated for cervical myofascial pain syndrome, only that refractory to conservative and minimally invasive treatments, with a positive diagnostic greater occipital nerve block and no structural cervical pathology, were included, resulting in a final sample of 14 patients.

All patients evaluated for CMPS were managed according to a structured (Figure 1), stepwise therapeutic algorithm. Most patients achieved satisfactory symptom control with first‐line conservative treatment, including therapeutic exercise and postural hygiene measures, and were therefore discharged without requiring further interventions.

Patients with persistent symptoms despite conservative management underwent second‐line treatments, such as interfascial injections or TrPs infiltrations. Cervical magnetic resonance imaging or other imaging studies were performed when clinically indicated to exclude structural cervical pathology. Only a small, highly selected subgroup of patients—those with refractory pain, occipital radiation, absence of relevant findings on imaging, and a positive but transient response to diagnostic GON blocks—were considered candidates for PRF treatment of the GON.

### 2.2. Intervention

All procedures were performed by experienced physicians under sterile conditions. Patients were placed prone, and ultrasound guidance was used to identify the GON at the interfascial plane between the obliquus capitis inferior and semispinalis capitis muscles [12, 13]. Following local anesthesia, a 22‐gauge radiofrequency needle was advanced to the target. Sensory stimulation confirmed proximity to the GON before delivering PRF (42°C for 4 min). No corticosteroids were administered.

### 2.3. Outcome Measures

Pain intensity was recorded using the Visual Analog Scale (VAS, 0–10). Measurements were collected before treatment and at follow‐up, documented in the clinical record. Adverse events were also recorded. VAS was selected as the primary outcome due to its widespread use in chronic pain studies and its sensitivity to clinically meaningful change.

### 2.4. Statistical Analysis

Data were analyzed using IBM SPSS v27 and R (4.5.2 version). Quantitative variables are presented as mean ± standard deviation (SD). Nonparametric tests were used to compare pre‐ and posttreatment VAS scores. Correlations between clinical variables were evaluated using Pearson or Spearman coefficients as appropriate. Significance was set at *p* < 0.05. Additionally, because of the lack of data, Bayesian analyses of the model were performed.

### 2.5. Ethics Approval

The study was approved by the Comité de Ética de la Investigación Provincial de Cádiz (CEIm), Spain (approval code SICEIA‐2024‐003,277, April 24, 2025). All patient data were anonymized before analysis in accordance with the EU General Data Protection Regulation (GDPR) and Spanish Law 3/2018.

## 3. Results

Fourteen patients (8 females, 6 males) met the inclusion criteria. The mean age was 46.8 ± 13.1 years (range 29–74) (Table 1). Most patients (93%) engaged in occupational or recreational physical activity. Analgesic use postprocedure was recorded in 92.9% of participants, and 28.6% had received prior TrP infiltrations.

**TABLE 1:** Demographic and clinical characteristics of patients.


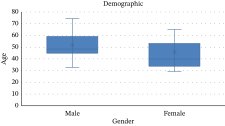

Pain intensity significantly decreased following PRF. Mean VAS scores improved from 7.93 (SD = 1.77) before treatment to 4.57 (SD = 2.38) after treatment. The mean reduction of 3.36 points surpassed the minimal clinically important difference for chronic pain interventions.

No significant correlations were observed between baseline pain and age, sex, or previous infiltrations. No major adverse events or neurological deficits were reported. Mild postprocedural soreness resolved within 24 h in all cases.

The Wilcoxon test revealed no differences in the grade of pain perception or response to treatment between gender groups. No differences could be determined in the range of time since improvement (measured in months until a new inquiry by the same question), whose mean was 77.6 months (SD ± 95.1, high dispersion). The same test (Wilcoxon) revealed statistically significant differences between pain outcome variable (VAS) pre‐ and postprocedure (*p* = 0.00375).

To evaluate the association between gender (the predictor) and postprocedure pain (the outcome), as well as VAS pre‐ and postprocedure (the outcomes), and age, we opted for Bayesian linear regression. This approach was selected for its robustness with small sample sizes (*N* = 14), enabling a more accurate quantification of uncertainty. Model convergence was assessed using the R‐hat statistic, with a value of 1.00 indicating successful convergence (model validity).

The women’s group had an estimated pain score of −0.77 points (95% CI: [−3.65, 2.07]) on the VAS scale compared with the men’s group. However, given that the 95% credibility interval is wide and includes zero, it cannot be concluded that there is a real difference between the groups.

The Bayesian model was then used to determine if the baseline VAS score (VAS‐pre) could predict the final outcome (VAS‐post). The analysis found no evidence that baseline pain was a significant predictor of the final postprocedure VAS score. The coefficient estimate (*β*) was −0.26, with a 95% credibility interval that included the null value [−1.07, 0.57]. Similarly, the influence of patient age on the VAS postprocedure score was evaluated.

The model did not find a statistically significant association. The estimated effect of age on VAS was zero (*β* = −0.00), with a 95% credibility interval centered on zero: [−0.12, 0.11].

## 4. Discussion

This study provides preliminary evidence supporting the potential role of PRF of the GON as a safe and effective treatment for CMPS. The observed mean reduction in pain intensity (3.36 points on the VAS) was both statistically and clinically significant. These findings are consistent with previous studies on PRF in occipital neuralgia and cervicogenic headache, including those by Navani et al. [18], Choi et al. [19], and Araujo Vázquez et al. [21]. Although direct comparisons are limited by differences in study design, patient populations, and outcome measures [22], the results support the clinical utility of PRF as a therapeutic option. In patients with refractory CMPS, PRF of the GON may be considered a last‐resort treatment, reserved for carefully selected individuals who have not responded to conservative management and diagnostic nerve blocks.

The neuromodulatory mechanism of PRF is believed to involve transient alterations in axonal membrane potential and synaptic transmission. Animal models suggest PRF upregulates c‐Fos and downregulates microglial activation in the dorsal horn, contributing to analgesia [9].

In our study, gender was not a significant predictor of postoperative pain perception. Bayesian analysis revealed considerable uncertainty in the effect estimate, suggesting that, with the available sample, a null effect cannot be ruled out. Similar findings were observed for age and preprocedure VAS scores. Although the estimated effect was −0.77, indicating a slight trend toward less pain in females, this result was not statistically significant. Low statistical power due to the small sample size likely limited our ability to detect a true difference. Future studies with larger cohorts are needed to confirm these findings.

Regarding the duration of improvement, the observation of an SD larger than the mean (95.1 months vs. 77.6 months) highlights the asymmetry of the sample. This variability represents a limitation of the retrospective follow‐up and underscores the need for prospective studies with standardized follow‐up protocols.

Despite the small sample size, the degree of pain relief observed supports the clinical relevance of PRF as an intermediate step between diagnostic nerve blocks [20] and more invasive procedures, showing promising results.

### 4.1. Limitations

This study has several limitations inherent to its retrospective observational design. The small sample size (*n* = 14), absence of a control group, and lack of standardized follow‐up duration limit causal inference and preclude definitive conclusions regarding the effectiveness of PRF of the GON.

The relatively low number of patients treated over 6 years reflects a strict, stepwise therapeutic algorithm in which PRF was reserved exclusively for patients with CMPS refractory to conservative therapies and minimally invasive interventions and without structural cervical pathology on imaging studies. While this approach reduced the number of eligible patients and limited statistical power, it closely reflects real‐world clinical practice in specialized pain and rehabilitation settings, potentially enhancing the external validity and clinical applicability of the findings.

Additionally, the retrospective nature of the study and variability in follow‐up duration may have contributed to heterogeneity in outcome measures, particularly regarding the duration of pain relief. The small sample size also limited the ability to identify predictors of treatment response or perform meaningful subgroup analyses.

Therefore, the present results should be interpreted as hypothesis‐generating. Future prospective studies, ideally randomized controlled trials with larger cohorts and standardized follow‐up protocols, are warranted to confirm these findings, optimize procedural parameters (including temperature, duration, and target depth), and better define patient selection criteria for PRF of the GON in CMPS.

Similar sample sizes and retrospective designs have been reported in previous studies evaluating radiofrequency‐based interventions for occipital and cervical pain syndromes, including pulsed and continuous radiofrequency of the GON [23–26]. This reflects the highly selected population eligible for these procedures and the exploratory phase of evidence generation in interventional pain medicine. Consequently, small cohorts are common in early clinical investigations of radiofrequency techniques [23–26], and the present study should be interpreted within this context as hypothesis‐generating rather than confirmatory.

## 5. Conclusion

Ultrasound‐guided PRF of the GON was associated with a clinically meaningful reduction in pain in a small cohort of patients with CMPS refractory to conventional therapy. The procedure appeared to be safe, minimally invasive, and well‐tolerated. These findings should be interpreted as hypothesis‐generating, and further prospective studies with larger samples and standardized follow‐up are required to confirm efficacy and define optimal patient selection.

## Funding

No external funding was received for this study.

## Ethics Statement

This study was approved by the Comité de Ética de la Investigación Provincial de Cádiz, Spain (SICEIA‐2024–003,277, April 24, 2025).

## Conflicts of Interest

The authors declare no conflicts of interest.

## Data Availability

The datasets analyzed are available from the corresponding author upon reasonable request.

## References

[1] Velasco M. , Dolor Musculoesquelético: Fibromialgia Y Dolor Miofascial, Revista Médica Clínica Las Condes. (2019) 30, no. 6, 414–427, 10.1016/j.rmclc.2019.10.002.

[2] Capó-Juan M. Á. , Síndrome De Dolor Miofascial Cervical: Revisión Narrativa Del Tratamiento Fisioterápico, Anales del Sistema Sanitario De Navarra. (2015) 38, no. 1, 105–115, 10.4321/s1137-66272015000100011. 25963463

[3] Villaseñor Moreno J. C. , Escobar Reyes V. H. , de la Lanza Andrade L. P. , and Guizar Ramírez B. I. , Síndrome De Dolor Miofascial. Epidemiologia, Fisiopatología, Diagnóstico Y Tratamiento, Revista de Especialidades Médico-Quirúrgicas. (2013) 18, no. 2, 148–157.

[4] Gallart Úbeda V. and Jordá L. M. , Diagnóstico Y Epidemiología Del Síndrome Miofascial, Asociación Andaluza Del Dolor Y Asistencia Continuada. Congreso. (2021) 5.

[5] Jin F. , Guo Y. , Wang Z. et al., The Pathophysiological Nature of Sarcomeres in Trigger Points in Patients With Myofascial Pain Syndrome: A Preliminary Study, European Journal of Pain. (2020) 24, no. 10, 1968–1978, 10.1002/ejp.1647. 32841448 PMC7693045

[6] Martínez Cuenca J. M. and Pecos Martín D. , Criterios Diagnósticos Y Características Clínicas De Los Puntos Gatillo Miofasciales, Fisioterapia. (2005) 27, no. 2, 65–81.

[7] Turo D. , Otto P. , Shah J. P. et al., Ultrasonic Characterization of the Upper Trapezius Muscle in Patients With Chronic Neck Pain, Ultrasonic Imaging. (2013) 35, no. 2, 173–187, 10.1177/0161734612472408. 23493615 PMC4332887

[8] Hila-Patiño M. , Munuera-Martínez P. V. , and Al-Shwely F. , Cervical Myofascial Pain. Statpearls, 2024, StatPearls Publishing.

[9] Lin W. , Lou L. , Chu D. , Lv Y. , Tian L. , and Wang B. , Efficacy of Pulsed Radiofrequency Stimulation in Patients With Chronic Pain: A Narrative Review, Front Pain Res (Lausanne). (2025) 6, 10.3389/fpain.2025.1544909. PMC12378335 40873698

[10] Appasamy M. , Lam C. , Alm J. , and Chadwick A. L. , Trigger Point Injections, Physical Medicine and Rehabilitation Clinics of North America. (2022) 33, no. 2, 307–333. 35526973 10.1016/j.pmr.2022.01.011 PMC9116734

[11] Urits I. , Schwartz R. H. , Patel P. et al., A Review of the Recent Findings in Minimally Invasive Treatment Options for the Management of Occipital Neuralgia, Neurol Ther. (2020) 9, no. 2, 229–241, 10.1007/s40120-020-00197-1. 32488840 PMC7606364

[12] Castillo-Rebolledo D. , Riveros A. , Sousa-Rodrigues C. F. , and Olave E. , Nervio Occipital Mayor: Trayecto, Relaciones Anatómicas E Implicancias Clínicas De Sus Posibles Sitios, International Journal of Morphology. (2017) 35, no. 3, 1129–1135.

[13] Cesmebasi A. , Muhleman M. A. , Hulsberg P. et al., Occipital Neuralgia: Anatomic Considerations, Clinical Anatomy. (2015) 28, no. 1, 101–108, 10.1002/ca.22468. 25244129

[14] Bentsianov B. and Blitzer A. , Facial Anatomy, Clinical Dermatology. (2004) 22, no. 1, 3–13, 10.1016/j.clindermatol.2003.11.011. 15158538

[15] Choi I. and Jeon S. R. , Neuralgias of the Head: Occipital Neuralgia, Journal of Korean Medical Science. (2016) 31, no. 4, 479–488, 10.3346/jkms.2016.31.4.479. 27051229 PMC4810328

[16] O″Neill F. , Nurmikko T. , and Sommer C. , Other Facial Neuralgias, Cephalalgia. (2017) 37, no. 7, 658–669, 10.1177/0333102417689995. 28133989

[17] Weiss C. , Meza N. , Rojo A. , and González-Hernández J. , Neuralgia Occipital (Arnold): Reporte De Dos Casos Y Revisión De La Literatura, Revista Memoriza.com. (2009) 3, 8–16.

[18] Navani A. , Mahajan G. , Kreis P. , and Fishman S. M. , A Case of Pulsed Radiofrequency Lesioning for Occipital Neuralgia, Pain Medicine. (2006) 7, no. 5, 453–456, 10.1111/j.1526-4637.2006.00217.x. 17014606

[19] Choi H. J. , Oh I. H. , Choi S. K. , and Lim Y. J. , Clinical Outcomes of Pulsed Radiofrequency Neuromodulation for the Treatment of Occipital Neuralgia, Journal of Korean Neurosurgical Society. (2012) 51, no. 5, 281–285, 10.3340/jkns.2012.51.5.281. 22792425 PMC3393863

[20] Gawel M. J. and Rothbart P. J. , Occipital Nerve Block in the Management of Headache and Cervical Pain, Cephalalgia. (1992) 12, no. 1, 9–13, 10.1046/j.1468-2982.1992.1201009.x. 1559261

[21] Araujo Vázquez M. , Lafuente Ojeda N. , Martínez Andreu F. J. et al., Radiofrecuencia Pulsada Para El Tratamiento De La Neuralgia Occipital: Análisis De Predictores Y Resultados, Revista de la Sociedad Española del Dolor. (2017) 24, no. 6, 356–358.

[22] Oliveira K. , Dhondt N. , Englesakis M. , Goel A. , and Hoydonckx Y. , Pulsed Radiofrequency Neuromodulation of the Greater Occipital Nerve for the Treatment of Headache Disorders in Adults: A Systematic Review, Canadian Journal of Pain. (2024) 8, no. 1, 10.1080/24740527.2024.2355571. PMC11195485 38915302

[23] Karaoğlan M. and Takmaz S. A. , Evaluating the Therapeutic Impact of Transcutaneous Radiofrequency Therapy on Myofascial Pain Syndrome Patients: A Clinical Study, Ankara Eğitim Ve Araştırma Hastanesi Tıp Dergisi. (2024) 57, no. 2, 64–68.

[24] Huang J. H. , Galvagno Jr S. M. , Hameed M. et al., Occipital Nerve Pulsed Radiofrequency Treatment: A Multi-Center Study Evaluating Predictors of Outcome, Pain Medicine. (2012) 13, no. 4, 489–497, 10.1111/j.1526-4637.2012.01348.x. 22390409

[25] Barnsley L. , Percutaneous Radiofrequency Neurotomy for Chronic Neck Pain: Outcomes in a Series of Consecutive Patients, Pain Medicine. (2005) 6, no. 4, 282–286, 10.1111/j.1526-4637.2005.00047.x. 16083457

[26] Turan S. A. , Aydın Ş. , and Can E. , Ultrasound-Guided Continuous Radiofrequency Ablation of the Proximal Greater Occipital Nerve is Effective in Refractory Occipital Neuralgia: A Retrospective Cohort Study, Arquivos De Neuro-Psiquiatria. (2025) 83, no. 03, 001–008, 10.1055/s-0045-1806813. PMC11964712 40174877

